# In Vitro Investigation of the Anti-Fibrotic Effects of 1-Phenyl-2-Pentanol, Identified from *Moringa oleifera* Lam., on Hepatic Stellate Cells

**DOI:** 10.3390/ijms25168995

**Published:** 2024-08-19

**Authors:** Watunyoo Buakaew, Sucheewin Krobthong, Yodying Yingchutrakul, Nopawit Khamto, Pornsuda Sutana, Pachuen Potup, Yordhathai Thongsri, Krai Daowtak, Antonio Ferrante, Catherine Léon, Kanchana Usuwanthim

**Affiliations:** 1 Department of Microbiology, Faculty of Medicine, Srinakharinwirot University, Bangkok 10110, Thailand; watunyoo@g.swu.ac.th; 2 Cellular and Molecular Immunology Research Unit (CMIRU), Faculty of Allied Health Sciences, Naresuan University, Phitsanulok 65000, Thailand; pornsudas64@nu.ac.th (P.S.); pachuenp@nu.ac.th (P.P.); yordhathait@nu.ac.th (Y.T.); kraid@nu.ac.th (K.D.); 3 Center of Excellence in Natural Products Chemistry (CENP), Department of Chemistry Faculty of Science, Chulalongkorn University, Bangkok 10330, Thailand; sucheewin.k@chula.ac.th; 4 National Center for Genetic Engineering and Biotechnology, NSTDA, Pathum Thani 12120, Thailand; yodying.yin@nstda.or.th; 5 Department of Chemistry, Faculty of Science, Chiang Mai University, Chiang Mai 50200, Thailand; nopawit_kh@cmu.ac.th; 6 Department of Immunopathology, South Australia (SA) Pathology, Women’s and Children’s Hospital, Adelaide, SA 5006, Australia; antonio.ferrante@adelaide.edu.au; 7 The Adelaide Medical School, The School of Biological Science and the Robinson Research Institute, University of Adelaide, Adelaide, SA 5000, Australia; 8 INSERM, UMR_S1255, Université de Strasbourg, Etablissement Français du Sang-GEST, 67000 Strasbourg, France; catherine.leon@efs.sante.fr

**Keywords:** 1-phenyl-2-pentanol, hepatic stellate cell, liver fibrosis, proteomics, *Moringa oleifera* Lam., molecular docking analysis

## Abstract

Liver fibrosis, characterized by excessive extracellular matrix deposition, is driven by activated hepatic stellate cells (HSCs). Due to the limited availability of anti-fibrotic drugs, the research on therapeutic agents continues. Here we have investigated *Moringa oleifera* Lam. (MO), known for its various bioactive properties, for anti-fibrotic effects. This study has focused on 1-phenyl-2-pentanol (1-PHE), a compound derived from MO leaves, and its effects on LX-2 human hepatic stellate cell activation. TGF-β1-stimulated LX-2 cells were treated with MO extract or 1-PHE, and the changes in liver fibrosis markers were assessed at both gene and protein levels. Proteomic analysis and molecular docking were employed to identify potential protein targets and signaling pathways affected by 1-PHE. Treatment with 1-PHE downregulated fibrosis markers, including collagen type I alpha 1 chain (*COL1A1*), collagen type IV alpha 1 chain (*COL4A1*), mothers against decapentaplegic homologs 2 and 3 (*SMAD2/3*), and matrix metalloproteinase-2 (*MMP2*), and reduced the secretion of matrix metalloproteinase-9 (MMP-9). Proteomic analysis data showed that 1-PHE modulates the Wnt/β-catenin pathway, providing a possible mechanism for its effects. Our results suggest that 1-PHE inhibits the TGF-β1 and Wnt/β-catenin signaling pathways and HSC activation, indicating its potential as an anti-liver-fibrosis agent.

## 1. Introduction

Liver fibrosis is the physiological consequence of the excessive wound healing process that responds to the liver injury [1]. Several etiological factors can cause the inflammation in the liver and increase the risk of fibrous scar formation, resulting from toxins, viral hepatitis, alcoholic liver disease (ALD), and non-alcoholic fatty liver disease (NAFLD) [2]. Characteristically, hepatic fibrosis is underpinned by the accumulation of extracellular matrix (ECM), especially collagen types I and III [3]. Several studies have revealed the key factors in the synthesis of ECM in response to hepatic injury include hepatic stellate cells (HSCs), which belong to hepatic mesenchymal cells [4,5,6,7]. In response to liver injury, transforming growth factor beta 1 (TGF-β1), secreted from the surrounding immune cells, induces fibrogenesis by stimulating HSCs, leading to myofibroblast differentiation. Activated myofibroblasts then release several proteins which are the key markers of hepatic fibrosis, including matrix metalloproteinases (MMPs), alpha-smooth muscle actin (α-SMA), and collagen type I, as well as specific phenotypic changes, such as fibrogenesis, contractility, and immunomodulation [3]. The process of hepatic fibrosis accumulation is reversible when the causes are removed. Progressive chronic liver inflammation can lead to the development of cirrhosis, the irreversible form of hepatic structural change, which affects liver function [8]. Since the specific medication for liver fibrosis remains limited there is a need to explore new agents to treat the condition.

Phytochemical active compounds have drawn attention in the field of anti-hepatic-fibrosis research since several active compounds isolated from plants have shown promising biological activities in vitro and in vivo [9,10,11,12,13]. *Moringa oleifera* Lam. (MO), a plant that belongs to the Moringaceae family [14], is one that has been extensively studied in various phytomedicine-related fields, for anti-inflammatory [15,16,17], anti-cancer [18,19,20], anti-microbial [21,22,23], and anti-hepatic-fibrosis activities [24,25]. The study from Wisitpongpun et al. showed that 1-phenyl-2-pentanol (1-PHE), the active compound isolated from the ethyl acetate extract of MO leaf, has anti-cancer activity by inducing cell cycle arrest and apoptosis in a triple-negative breast cancer cell line [26]. This compound has been used as a food additive and a flavoring agent [27]. However, the anti-hepatic-fibrosis activity of the 1-PHE remains unexplored.

In this study, we aimed to evaluate the anti-liver-fibrotic activity of 1-PHE in the TGF-β1-induced HSC model. The expression of activated HSC markers was measured at the gene and protein levels. Proteomics analysis was employed to visualize the proteins that might be involved in the mechanism of action of 1-PHE. Moreover, molecular docking analysis was used to predict the potential binding interaction between the active compound and candidate protein targets. Our findings provided the valuable insights into the potential anti-fibrotic agent of 1-PHE isolated from MO plants, which contributes to the growing knowledge on finding novel anti-hepatic-fibrosis agents.

## 2. Results

### 2.1. The Effects of Crude MO Extract and 1-PHE on Cell Viability

**Figure 1:**
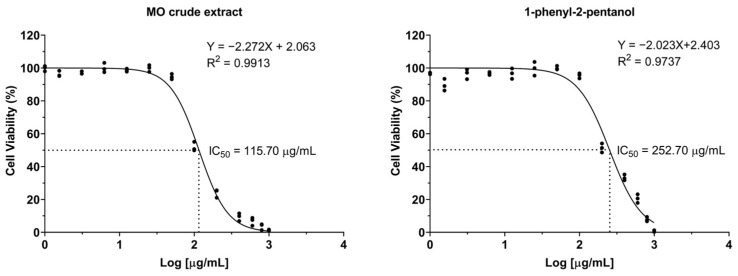
The dose–response curves of LX-2 cell viability after treatment with varying concentrations of crude Moringa extract or 1-phenyl-2-pentanol.

LX-2 cells were exposed to varying concentrations of crude MO extract and 1-PHE for 24 h, and then examined for cell viability by the resazurin dye exclusion test. Dose–response curves were analyzed using GraphPad Prism’s non-linear regression tools (Figure 1). The formulas to calculate IC_5_ and IC_10_ values are shown below [28], where F represents the desired inhibition percentage and H is the Hill slope. The IC_5_, IC_10_, and IC_50_ of crude MO extract were 31.64, 43.95, and 115.61 µg/mL, respectively. In contrast, 1-PHE showed less toxicity for LX-2 cells than the crude MO extract, with IC_5_, IC_10_, and IC_50_ values of 59.00, 85.37, and 252.92 µg/mL, respectively. Based on these results, concentrations of 40 µg/mL (crude extract) and 80 µg/mL (1-PHE) were chosen as the upper limits for further investigations.

(1) $${IC}_{F}={\left(\frac{F}{100-F}\right)}^{\sqrt{H}}\cdot {IC}_{50}$$

### 2.2. The Effects of Crude MO Extract and 1-PHE on Expression of Liver Fibrotic Markers

**Figure 2:**
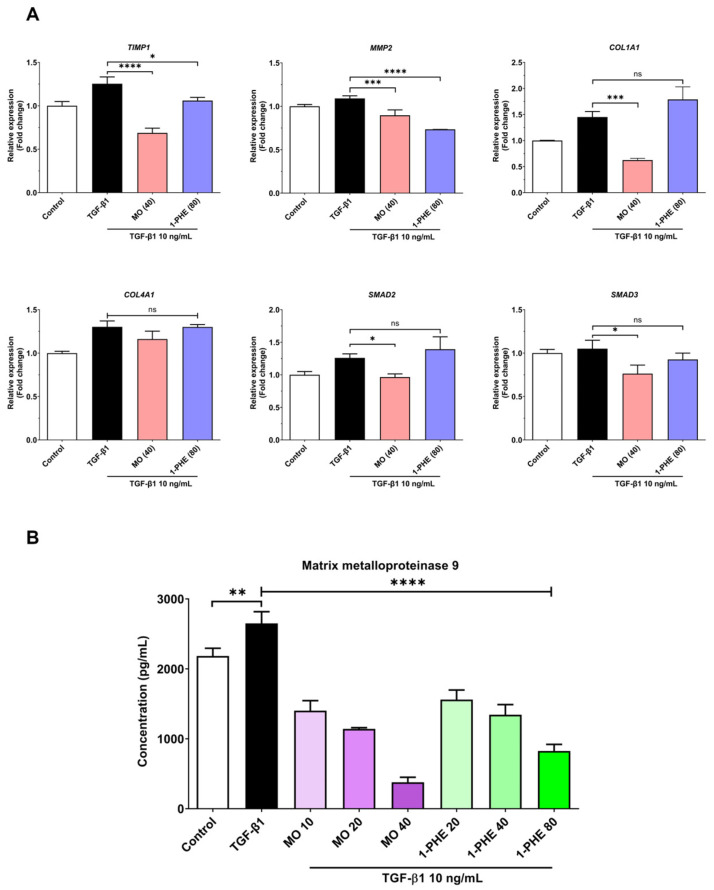
The expression of liver fibrotic-associated genes and the MMP-9 level in crude MO extract and 1-PHE treated cells. The LX-2 cells at 2 × 10^5^ cells/well were incubated with different concentrations of crude MO extract or 1-PHE in the presence of 10 ng/mL TGF-β1 for 48 h. The cells were harvested and the mRNA expression measured using real-time qRT-PCR. (**A**) The candidate liver fibrotic-associated genes, as shown above, include *COL1A1*, *TIMP1*, *MMP2*, *SMAD2*, and *SMAD3*. The relative gene expression was normalized to *GAPDH*. (**B**) The cell culture supernatant was collected and evaluated for the MMP-9 level. The data are presented as mean ± SD. *p*-value < 0.0332 (*), *p*-value < 0.0021 (**), *p*-value < 0.0002 (***), *p*-value < 0.0001 (****).

The expression of selected liver fibrosis markers (both gene expression and MMP-9 levels) in LX-2 cells exposed to varying concentrations of crude MO extract and 1-PHE was evaluated using qRT-PCR and ELISA. Cells were treated for 48 h with 10 ng/mL TGF-β1. Crude MO significantly suppressed the expression of *COL1A1*, *TIMP1*, *MMP2*, *SMAD2*, and *SMAD3* genes in the TGF-β1-treated LX2 cells. High-dose 1-PHE treatment significantly inhibited *TIMP1* and *MMP2* expression (Figure 2A). Both crude MO extract and 1-PHE significantly reduced the release of MMP-9 in a dose-dependent manner (Figure 2B). These results suggest potential anti-fibrotic activity of crude MO extract and 1-PHE at both transcriptional and protein levels.

### 2.3. Proteomic Analysis of 1-PHE Treatment in LX-2 Cells

**Figure 3:**
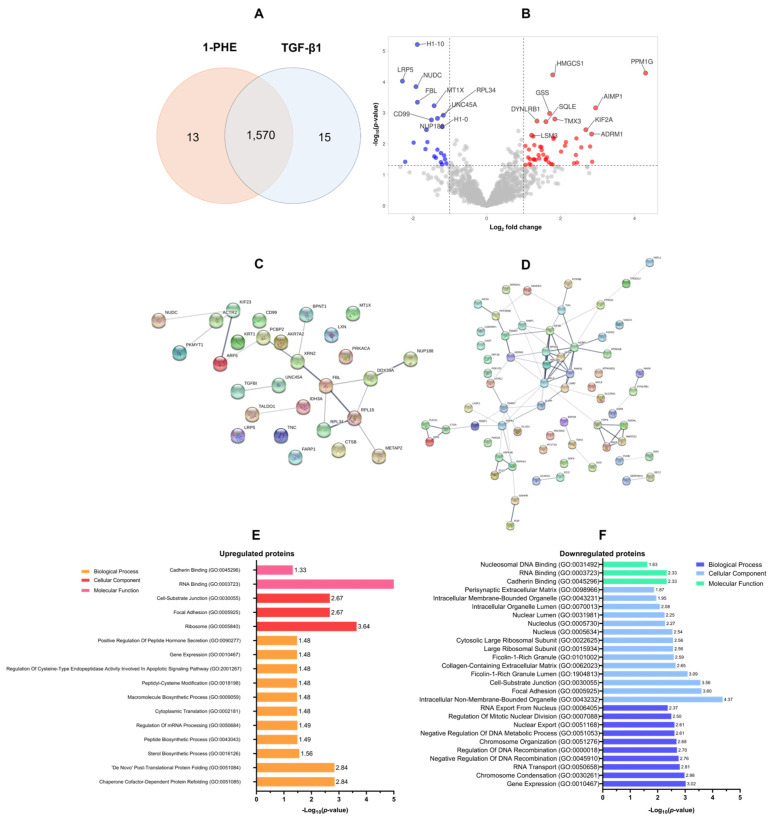
The proteomic profiling of DEPs and GO annotation analysis. In total, 1570 DEPs were identified following treatment of LX-2 cells with 1-PHE and TGF-β1 (**A**). Statistical thresholds (log_2_ fold change ≤1.5 or ≥1.5; *p*-value < 0.05) were applied to distinguish significantly upregulated (n = 68; red dots) and downregulated (n = 30; blue dots) proteins (**B**). Protein–protein interaction (PPI) network analysis demonstrated interconnectedness within the sets of upregulated and downregulated DEPs (**C**,**D**). To illuminate the potential functions of these DEPs, Gene Ontology (GO) annotation was employed, categorizing proteins by biological process, cellular component, and molecular function (**E**,**F**).

A proteomic analysis was conducted to evaluate the effects of 1-PHE treatment on TGF-β1 LX-2 cells for 48 h. Statistical analysis (*p*-value < 0.05 and log_2_ fold change) revealed differential expression of 1570 proteins (DEPs). Treatment with 1-PHE resulted in the upregulation of 68 proteins and the downregulation of 30 proteins (Figure 3A,B). Table 1 summarizes the top 10 most significantly impacted proteins. A protein–protein interaction (PPI) network analysis, constructed using a stringent interaction score threshold (0.700), revealed significant interconnectivity among differentially expressed proteins (Figure 3C,D). This suggests potential functional relationships among the upregulated and downregulated proteins.

Gene Ontology (GO) annotation demonstrated that the upregulated DEPs were primarily involved in chaperone-mediated protein refolding (GO:0051085 and GO:0051084), as well as sterol biosynthesis (GO:0016126). Conversely, downregulated DEPs were associated with gene expression regulation (GO:0010467), the process of chromosome condensation (GO:0030261), and RNA transport (GO:0050658). Analysis of cellular localization showed that upregulated proteins were found in the ribosome (GO:0005840), focal adhesions (GO:0005925), and cell–substrate junctions (GO:0030055). In contrast, downregulated DEPs were predominantly associated with non-membrane-bound intracellular organelles (GO:0043232), focal adhesions (GO:0005925), cell–substrate junctions (GO:0030055), ficolin-1-rich granule lumens (GO:1904813), and the collagen-containing extracellular matrix (GO:0062023). Molecular function classification revealed that upregulated DEPs exhibited a strong propensity for RNA binding (GO:0003723), cadherin binding (GO:0045296), and protein homodimerization (GO:0042803). Downregulated DEPs also demonstrated cadherin- and RNA-binding activities, as well as an affinity for nucleosomal DNA binding (GO:0031492), as shown in Figure 3E,F.

**Figure 4:**
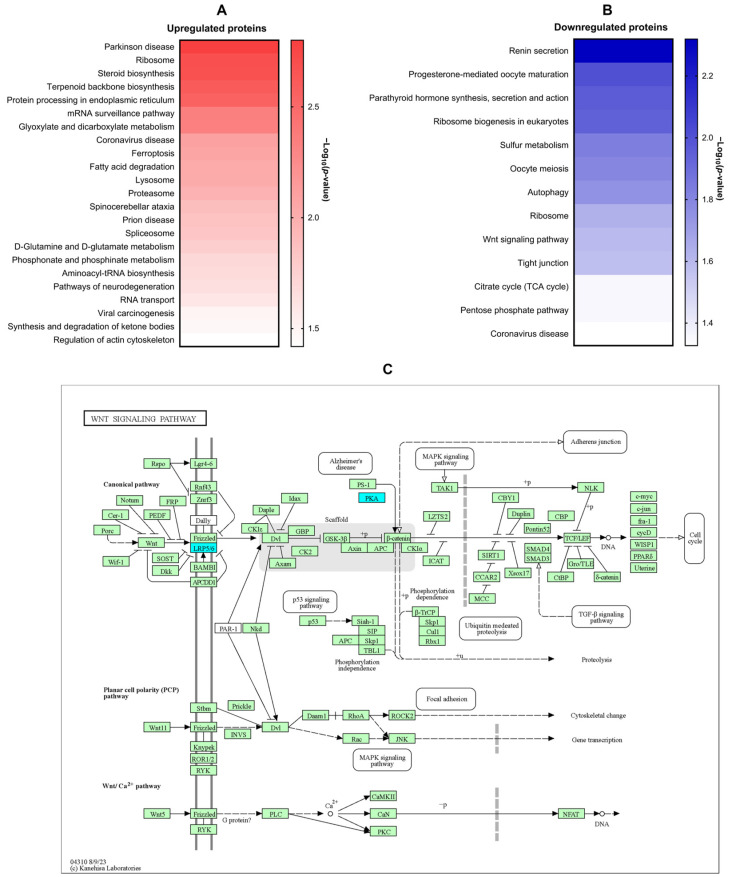
KEGG signaling pathway enrichment analysis of DEPs. KEGG signaling pathway enrichment analysis was performed on DEPs to elucidate the potential impact of 1-PHE on signaling pathways within LX-2 cells. Notably, Parkinson’s disease emerged as the most significantly enriched pathway associated with upregulated DEPs (**A**). In contrast, the renin secretion signaling pathway demonstrated the strongest enrichment among downregulated proteins (**B**). Intriguingly, the Wnt signaling pathway, a pathway strongly implicated in liver fibrosis and HSC activation, was also found to be downregulated (**C**). Downregulated genes were indicated by the color blue, while other genes within the pathway were represented by green. *LRP5*, LDL-receptor-related protein 5; *PRKACA*, protein kinase cAMP-activated catalytic subunit alpha.

KEGG enrichment analysis revealed differential modulation of several signaling pathways in LX-2 cells following 1-PHE treatment. Notably, pathways associated with Parkinson’s disease, ribosomal function, steroid and terpenoid backbone biosynthesis, and endoplasmic reticulum protein processing were significantly upregulated. In contrast, pathways related to renin secretion, eukaryotic ribosome biogenesis, autophagy, Wnt signaling, and tight junction formation were downregulated (Figure 4A,B). To highlight pathways implicated in hepatic fibrosis [29,30,31,32], the Wnt signaling pathway was diagrammed (Figure 4C). Within this pathway, downregulated genes, LDL-receptor-related protein 5 (*LRP5*) and protein kinase cAMP-activated catalytic subunit alpha (*PRKACA*), are indicated in cyan.

### 2.4. Molecular Docking Analysis of Candidate Target Proteins

**Figure 5:**
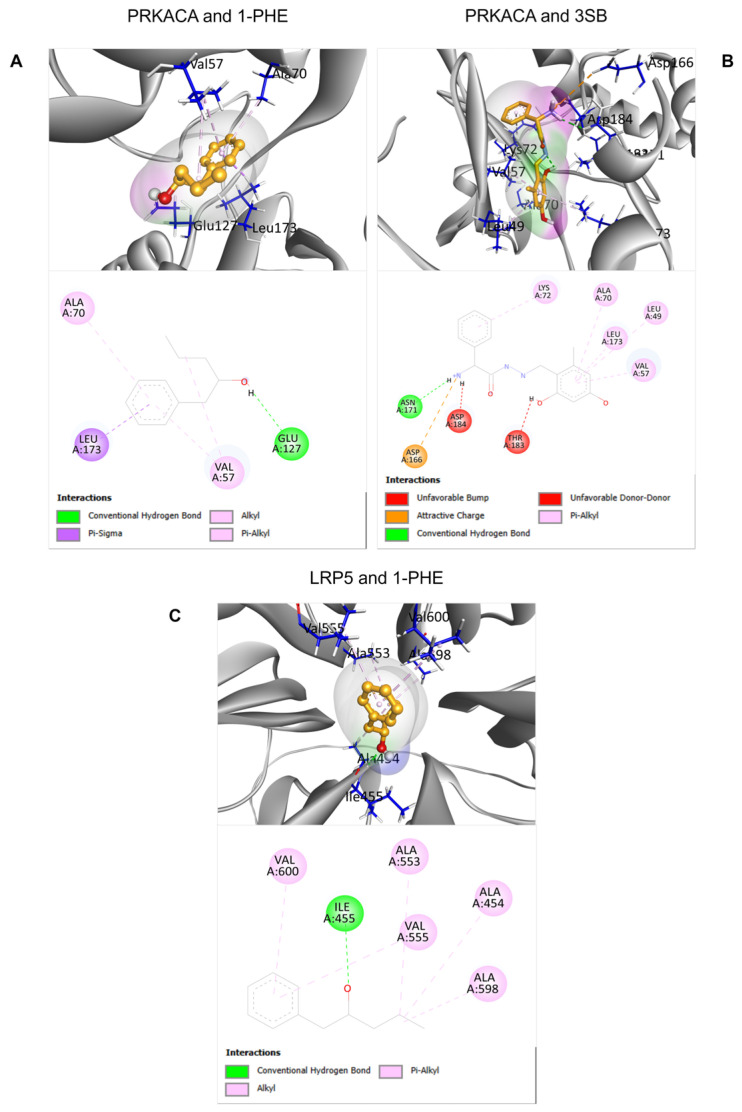
The 2D and 3D structural characteristics of ligand-protein complexes. Specifically, the interactions between PRKACA and two ligands, 1-PHE and 3SB, were visualized (**A**,**B**). Additionally, the interaction between 1-PHE and LRP5 was investigated (**C**).

To predict potential interactions between 1-PHE and proteins within the Wnt signaling pathway, molecular docking analyses were performed on two downregulated proteins identified through proteomic analysis: PRKACA and LRP5. For PRKACA, the binding score of 1-PHE (−6.033) was comparable to that of the known binding compound 3SB (−6.306), suggesting a potential binding affinity of 1-PHE for the same binding site on PRKACA. In the case of LRP5, due to the absence of a known binding compound and a corresponding experimental crystal structure in the RCSB PDB database, the docking site was determined solely based on protein cavity identification using the BIOVIA Discovery Studio Visualizer software version 21.1.0.20298 (Dassault Systèmes, San Diego, CA, USA). Despite these limitations, the calculated binding score between 1-PHE and LRP5 (−6.055) indicated a favorable interaction, as detailed in Table 2. Figure 5 provides in-depth 2D and 3D visualizations of the predicted binding poses and specific chemical interactions between 1-PHE and both target proteins.

### 2.5. Molecular Dynamics Simulation

**Figure 6:**
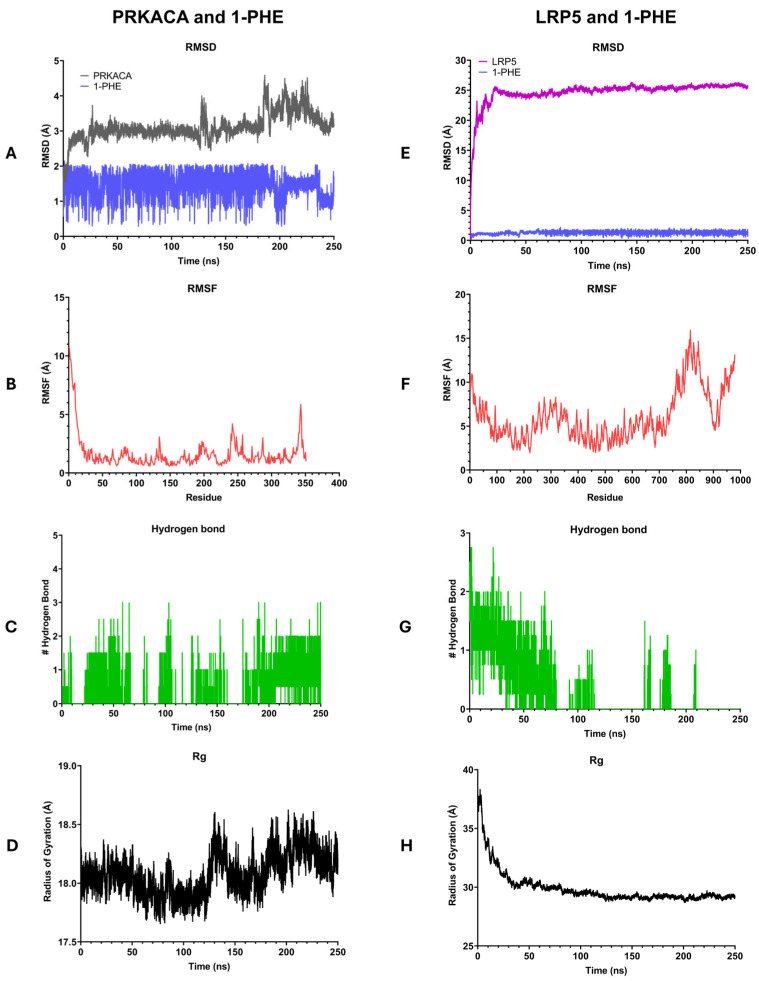
Illustration of the results of 250-nanosecond molecular dynamics simulations comparing the interactions of 1-PHE with PRKACA (**A**–**D**) and LRP5 (**E**–**H**). (**A**,**D**) present root mean square deviation (RMSD) plots, (**B**,**F**) display root mean square fluctuation (RMSF) plots, (**C**,**G**) depict numbers of hydrogen bonds, and (**D**,**H**) showcase radius of gyration (Rg) plots.

Molecular dynamics simulations were conducted to investigate the interactions between 1-PHE and two target proteins, PRKACA and LRP5, as depicted in Figure 6. During 250 ns, the 1-PHE bound stably within the binding site of both proteins. The stability of the formed complexes was assessed by calculating the root mean square deviation (RMSD). The PRKACA-1-PHE complex exhibited a stable RMSD of 2–5 Å over the 250 ns simulation (Figure 6A), indicative of a robust complex formation. In contrast, the LRP5-1-PHE complex demonstrated initial fluctuations in RMSD before stabilizing (Figure 6E). After 30 ns, both protein–ligand complexes reached equilibrium as indicated by the RMSD of protein less than 2 Å. To evaluate the flexibility of the complexes, root mean square fluctuations (RMSFs) were computed. The PRKACA-1-PHE complex displayed lower RMSF values compared to the LRP5-1-PHE complex (Figure 6B,F), suggesting greater rigidity in the former. Hydrogen bond analysis revealed a higher number of hydrogen bonds in the PRKACA-1-PHE complex (Figure 6C) compared to the LRP5-1-PHE complex (Figure 6G), further supporting the notion of a more stable interaction. The radius of gyration (Rg) was employed to assess the compactness of the complexes. The PRKACA-1-PHE complex maintained a stable Rg of 18.5 Å throughout the simulation (Figure 6D), signifying a compact structure. Conversely, the LRP5-1-PHE complex exhibited a higher initial Rg value that gradually decreased over time (Figure 6H), suggesting a more compact conformation. Collectively, the MD simulation results indicate that 1-PHE can form a stable complex with PRKACA and LRP5. However, experimental binding assays are necessary to corroborate these computational findings.

## 3. Discussion

Hepatic stellate cells (HSCs) are recognized as central mediators of extracellular matrix (ECM) production in hepatic fibrosis. Upon pathological stimuli associated with liver fibrosis, this phenotypic shift is orchestrated by a complex network of growth factors and signaling cascades, including TGF-β/SMAD, PDGF, NF-κB, and Wnt/β-catenin [1,3,4,7,33]. Of these, the TGF-β/SMAD pathway stands as the most extensively investigated, with TGF-β1 binding to its cognate receptor (TGF-β receptor 1), initiating a cascade of downstream effectors [34,35]. The activation of the TGF-β/SMAD signaling pathway in HSCs resulted in the upregulation of several key genes associated with hepatic fibrosis, including *TIMP1*, *SMAD2*, *SMAD3*, *COL1A1*, and *MMP2*, as well as increased secretion of MMP-9 protein (Figure 2). Treatment with the crude extract of MO mitigated the expression of these fibrosis-associated markers at both the mRNA and protein levels. This suggests that MO extract may inhibit HSC activation by interfering with the TGF-β/SMAD signaling cascade, particularly targeting the production of *SMAD2* and *SMAD3* transcription factors. In contrast, treatment with 1-PHE significantly reduced the mRNA expression of *TIMP1* and *MMP2*, along with MMP-9 secretion, indicating that 1-PHE may operate through an alternative signaling pathway involved in the production of MMPs, distinct from the TGF-β/SMAD pathway.

Proteomic analysis of 1-PHE-treated, activated LX-2 cells demonstrated alterations in multiple signaling cascades. Notably, proteins integral to the Wnt/β-catenin pathway exhibited downregulation, including low-density-lipoprotein-receptor-related protein 5 (LRP5) and protein kinase cAMP-activated catalytic subunit alpha (PRKACA). Several studies have suggested that the Wnt/β-catenin pathway is implicated in HSC activation [29,30,31,32,33]. Activation of this pathway commences with β-catenin binding to its receptor, LRP5/6, triggering the integrin-adhesion complex (IAC) pathway through integrin-mediated sensing of mechanical cues. The observed decrease in LRP5 protein expression following 1-PHE treatment raises the possibility an interference with the Wnt/β-catenin signaling cascade. This may occur through 1-PHE suppressing receptor expression. Nevertheless, additional investigations are warranted to validate this concept and clarify the precise mechanism by which 1-PHE disrupts the Wnt/β-catenin signaling cascade during HSC activation.

Molecular docking analysis and molecular dynamics simulations were conducted to explore potential interactions between 1-PHE and the Wnt/β-catenin signaling pathway proteins PRKACA and LRP5. Comparative binding energy analysis using Autodock Vina within the Chimera software platform version 1.18 revealed similar binding affinities between 1-PHE and 3SB at the same binding site on PRKACA (Table 2). These findings suggest that 1-PHE may bind to PRKACA at a site overlapping with that of a known ligand. However, the possibility of alternative binding sites on PRKACA cannot be excluded. For LRP5, in the absence of experimental structural data and a known ligand-binding site, a potential binding site was predicted using BIOVIA Discovery Studio Visualizer software version 21.1.0.20298. The calculated binding score of 1-PHE to LRP5 was comparable to that for PRKACA. Molecular dynamics simulations, as depicted in Figure 6, demonstrated the sustained stability and compact conformation of the 1-PHE complexes with both PRKACA and LRP5 over a 250-nanosecond timescale. Notably, the complex formed with PRKACA exhibited superior stability compared to that with LRP5. Based on these in silico results, we hypothesize that 1-PHE could disrupt β-catenin signaling by interacting with PRKACA and LRP5, ultimately attenuating HSC activation. It is crucial to emphasize that these predictions are based solely on computational modeling and require experimental binding validation to confirm potential target–ligand interactions.

The MMPs are integral to the activation of HSCs during liver fibrosis. MMP-2 and MMP-9 are particularly crucial in the remodeling of the ECM during hepatic fibrogenesis [36,37]. Research indicate that the Wnt/β-catenin pathway is involved in MMP production [38,39,40]. The proteomic analysis and MMP expression data following 1-PHE treatment suggest that the observed decrease in *MMP2* mRNA and MMP-9 secretion might be due to interference with the Wnt/β-catenin pathway. This interference may attenuate the TGF-β1-mediated activation of HSCs, potentially offering a therapeutic strategy for mitigating hepatic fibrosis [41].

Our finding is the first to show that 1-PHE may suppress HSC activation induced by TGF-β1, possibly through modulation of the Wnt/β-catenin signaling pathway, given that inhibition of Wnt/β-catenin signaling is a recognized therapeutic strategy for liver fibrosis [29,30]. This study positions 1-PHE as a promising candidate for further investigation in the development of novel anti-fibrotic agents. However, in vivo and clinical studies are essential to validate these findings and to fully elucidate the pharmacological effects and mechanism of action of 1-PHE in the context of liver fibrosis.

## 4. Materials and Methods

### 4.1. Chemicals and Reagents

Dulbecco’s modified Eagle’s medium (DMEM), fetal bovine serum (FBS), antibiotic–antimycotic, phosphate-buffered saline (pH 7.4), and trypsin-EDTA (0.25%) were purchased from Gibco (Thermo Fisher Scientific, Waltham, MA, USA). Human recombinant TGF-beta 1 (TGF-β1) was obtained from STEMCELL Technologies (STEMCELL Technologies Canada, Inc., Vancouver, BC, Canada). Dimethyl sulfoxide (DMSO) was purchased from VWR International (VWR Corporate Headquarters, West Chester, PA, USA). Polysorbate 80 (Tween 80) was purchased from BioBasic (BioBasic, Inc., Markham, ON, Canada). Human matrix metalloproteinase 9 (MMP-9) ELISA kit was obtained from Sino Biological (Sino Biological, Inc., Beijing, China). Resazurin sodium salt was purchased from Sigma-Aldrich (MilliporeSigma, Burlington, MA, USA). Trizol reagent and Pierce BCA protein assay kits were obtained from Invitrogen and Thermo Scientific^™^, respectively (Thermo Fisher Scientific, Waltham, MA, USA). Tetro^™^ cDNA Synthesis and SensiFAST^™^ SYBR^®^ No-ROX kits were purchased from Meridian Bioscience (Meridian Diagnostics & Corporate Offices, Cincinnati, OH, USA). Protease-phosphatase inhibitor cocktail was purchased from Cell Signaling Technology (Cell Signaling Technology, Inc., Danvers, MA, USA). RapidGest SF was obtained from Waters^™^ (Waters Corporation, Milford, MA, USA). Trypsin and Tris(2-carboxyethyl) phosphine (TCEP) were purchased from Promega (Promega Corporation, Madison, WI, USA). Iodoacetamide (IAA) was purchased from GE Healthcare Technologies (GE HealthCare Technologies, Inc., Chicago, IL, USA). LC-MS/MS analysis reagents, including acetonitrile, formic acid, ammonium bicarbonate, acetone, and water were purchased from J.T.Baker (Fisher Scientific, Houston, TX, USA).

### 4.2. Cell Line and Culture

The immortalized human hepatic stellate cell line, LX-2, was kindly provided by associate professor Dr. Saranyapin Potikanond (Department of Pharmacology, Chiang Mai University, Thailand). The cells were cultured in high-glucose Dulbecco’s modified Eagle’s medium (DMEM) supplemented with 4 mM L-glutamine, 2% fetal bovine serum (FBS), and 1% antibiotic–antimycotic in a 37 °C, 5% CO_2_ humidified incubator. The subculture of the cells was conducted after approximately 80% confluence of the cells was reached using trypsin-EDTA (0.25%) reagent.

### 4.3. Preparation of MO Leaf Crude Extract and Active Compound

The protocol of extraction and identification of active compound, 1-phenyl-2-pentanol (1-PHE), from the *Moringa oleifera* Lam. (MO) leaf was performed as previously describe in [26]. The crude extract from ethyl acetate, which represented the highest activity of inhibiting cancer cell growth, was used to evaluate the anti-liver-fibrosis in this study. 1-phenyl-2-pentanol (Sigma Aldrich, St. Louis, MO, USA), the active compound, was purchased for use in this study. The crude extract and active compound were prepared in dissolving solvent (DMSO:Tween 80) (1:1) to improve the solubility in water. The final residue percentage of the dissolving solvent in the cell culture experiments was less than 0.5% in the highest concentration of both compounds.

### 4.4. Cytotoxicity Assessment by Resazurin Reduction Assay

The optimal concentrations of crude MO extract and 1-PHE for LX-2 experiments were evaluated by resazurin reduction assay [42]. The LX-2 cells (2 × 10^4^ cells/well) were plated in 96-well cell culture plate. The cells were treated with differential diluted concentrations (0–1000 µg/mL) of crude extract and 1-PHE. Then, the resazurin reagent (at final concentration 25 µg/mL) was added to the cell mixture and incubated in the incubator for 24 h. The fluorescence signal was measured using an EnSpire^®^ microplate reader (Petro Emphor Co. W.L.L., Doha, Qatar) with a 560 nm excitation/590 nm emission filter set. The inhibitory concentration (IC) was calculated using GraphPad Prism version 8.0.1 (GraphPad Software, Boston, MA, USA). The IC_10_ (concentration inhibiting 10% cell viability) of each compound was used as the maximum concentration for further experiments.

### 4.5. Real-Time Quantitative Reverse Transcription PCR

The expression of fibrotic marker genes in LX-2 cells, including *COL1A1*, *COL4A1, TIMP1*, *SMAD2*, *SMAD3*, and *MMP2*, was measured by real-time qRT-PCR. Briefly, the cells were treated with the mixture of TGF-β1 (10 ng/mL) and crude MO extract or 1-PHE for 24 h. The mRNA in each condition was extracted using Trizol reagent, as described in the manufacturer’s protocol. The total RNA was converted to cDNA using a Tetro^™^ cDNA Synthesis Kit. The PCR reaction was conducted using a SensiFAST^™^ SYBR^®^ No-ROX Kit. The thermal cycler settings were composed of polymerase activation (95 °C, 1 min), followed by 45 cycles of denaturation (95 °C, 10 s) and annealing/extension (60 °C, 1 min) on the CFX96 Touch Real-Time PCR Detection System (Bio-Rad Laboratories, Hercules, CA, USA). The oligonucleotide primers used in this study are shown in Table 3 [43].

### 4.6. Enzyme-Linked Immunosorbent Assay (ELISA)

To measure the level of MMP-9 secretion, which is one of the key markers in fibrogenesis response of LX-2 cells, the cells culture supernatant from the experiment was collected and the human matrix metalloproteinase 9 ELISA kit was used according to the manufacturer’s protocol. The absorbance of the ELISA reaction was measured at wavelength 450 nm using an EnSpire^®^ microplate reader.

### 4.7. Sample Preparation for LC-MS/MS Analysis

LX-2 cells treated with 1-PHE for 48 h were harvested. The cells were washed twice with 1X PBS (pH 7.4). Total protein was extracted using lysis buffer (1X RIPA with protease–phosphatase inhibitors) and incubated on ice for 5 min. The lysate was sonicated (5 cycles, 30 s each, with rests on ice) and centrifuged (14,000× *g*, 4 °C, 10 min). The protein concentration was determined using a Pierce™ BCA protein assay kit following the manufacturer’s instructions. According to the previous sample preparation protocol with minor modifications [44], the protein concentration was achieved using a 3 kDa molecular weight cutoff filter, followed by precipitation with ice-cold acetone (1:5 *v*/*v*). The pellet was resuspended in 0.3% RapidGest SF/2.5 mM ammonium bicarbonate. A 30 µg aliquot of protein underwent tryptic digestion. Disulfide bonds were reduced (1 mM tris(2-carboxyethyl) phosphine, TCEP, 37 °C, 2 h) and alkylated (5 mM iodoacetamide, IAA, room temperature, 50 min, protected from light). The sample was desalted (Zeba Spin Column) (Thermo Fisher Scientific, Waltham, MA, USA) prior to a second trypsin digestion (1:40 enzyme–protein ratio, 37 °C, 6 h). After drying, the digested peptides were resuspended in 0.1% formic acid for LC-MS/MS analysis.

### 4.8. LC-MS/MS Setting and Data Processing for Proteomic Analysis

Samples were analyzed via liquid chromatography–tandem mass spectrometry (LC-MS/MS) (HF-X hybrid Quadrupole-Orbitrap, EASY-nLC1000 system, nano C18 column) (Thermo Fisher Scientific, Waltham, MA, USA) operating in positive ionization mode. Chromatographic separation employed a 3–60% gradient of 90% acetonitrile/0.1% formic acid over 135 min (300 nL/min), using 0.1% formic acid in water as mobile phase A. Column regeneration and re-equilibration steps were performed. Data-dependent acquisition (TopN15) was carried out with higher-energy collisional dissociation (29 eV)-guided peptide analysis. MS parameters and database search (UniProt Homo sapiens, 14 January 2023) were implemented in Proteome Discoverer™ 2.4 software (Thermo Fisher Scientific, Waltham, MA, USA). Peptide/protein tolerances, modifications, and a 1% FDR were applied. Data were normalized (total intensity count) and subjected to pathway analysis via PADOG in Reactome v84 (*Homo sapiens*, 25 February 2023) [45].

### 4.9. Bioinformatic Analysis of Proteomic Data

We used several bioinformatic tools to further investigate the differentially expressed proteins (DEPs) identified using Proteome Discoverer™ 2.4 software. DEPs in the treatment group were filtered based on a log2 fold change threshold (≤−1.5 and ≥1.5) and an adjusted *p*-value of <0.05. Visualization of DEPs was achieved through a volcano plot generated using VolcaNoseR2 [46] https://huygens.science.uva.nl/VolcaNoseR2/ (accessed on 10 February 2024). Cytoscape software version 3.10.1 [47] with the aid of stringApp application version 2.0.3 [48] was employed to analyze and visualize the protein–protein interaction network within the DEPs. Enrichr web server [49,50,51] https://maayanlab.cloud/Enrichr/ (accessed on 10 February 2024). provided Gene Ontology (GO) annotations, classifying DEPs by biological process, cellular component, and molecular function. The Kyoto Encyclopedia of Genes and Genomes (KEGG) database [52,53,54] was used to map DEPs onto signaling pathways, facilitating pathway enrichment analysis.

### 4.10. Molecular Docking Analysis

The crystal structures of candidate proteins identified from the proteomics, including low-density-lipoprotein-receptor-related protein 5 (LRP5) and the catalytic subunit alpha of cAMP-dependent protein kinase (PRKACA), were retrieved from public databases (LRP5-AlphaFold protein structure database [55,56], PRKACA-RCSB PDB database (PDB ID: 3OXT) [57,58]). Chemical structures of 1-PHE (CID: 8842) and the known binding ligand of PRKACA, 3SB ((2s)-2-amino-N’-[(1e)-(2,4-dihydroxy-6-methylphenyl)methylidene]-2-phenylethanehydrazide; CID: 137348168), were obtained from PubChem [27]. Standard preparation procedures were applied to both the target proteins and ligands using the Dock Prep tool [59] within UCSF Chimera alpha version 1.18 [60]. This involved removing non-standard residues and water molecules, assigning charges, and modifying incomplete amino acid side chains. Docking simulations were then performed using AutoDock Vina software version 1.2.3 [61,62]. BIOVIA Discovery Studio Visualizer version 21.1.0.20298 was employed to analyze 2D and 3D interactions between target proteins and the compound.

### 4.11. Molecular Dynamics (MD) Simulation

Molecular dynamics (MD) simulations of 1-PHE in complex with LRP5 and PRKACA proteins were executed using the GROMACS 2022.4 software package [63] on GPU accelerators. The simulation protocol employed in this study was adapted from a previously published method [64] with minor adjustments. The protein topology was prepared using the *gmx_pdb2gmx* module and was parameterized using the AMBER ff99SB force field. The ligand structure was parameterized using the general AMBER force field (GAFF). The charge of 1-PHE was calculated using the AM1-BCC method using the acpype package. The protein–ligand complexes were solvated in a TIP3P water box with a 12 Å buffer distance using the tleap utility. The system was neutralized with Na^+^ and Cl^−^ ions to a concentration of 0.15 M. The system was initially energy minimized using the steepest descent algorithm to a tolerance of 10 kJ/mol/nm. Equilibration was conducted in two stages: a constant number of particles, volume, and temperature (NVT), followed by a constant number of particles, pressure, and temperature (NPT), ensembles for 1000 ps each. Production simulations were run for 250 ns at 310 K and 1 bar, with coordinates saved every 10 ps. The MD trajectories were analyzed using GROMACS tools. The dynamics of the system was monitored using by the root mean square deviation (RMSD) and root mean square fluctuation (RMSF) using the *gmx_rms* and *gmx_rmsf* utilities, respectively. Hydrogen bond analysis was conducted with *gmx_hbond* and visual molecular dynamics (VMD) [65], with the following criteria: distance between donor and acceptor ≤3.5 Å and an angle cutoff value of 20°. The GROMOS method was performed for clustering analysis of the protein backbone, with a cutoff of 1.5 Å.

### 4.12. Statistical Analysis

To analyze differences in means across groups, a one-way analysis of variance (ANOVA) was employed followed by Tukey’s post hoc test for multiple comparisons in GraphPad Prism software version 8.0.1 (GraphPad Software, Boston, MA, USA). Results were reported as mean ± standard deviation (SD), with statistical significance determined at a *p*-value threshold < 0.05.

## 5. Conclusions

These findings indicate that 1-PHE may attenuate HSC activation induced by TGF-β1 via multiple mechanisms. This is evidenced by the observed suppression of established hepatic fibrosis markers, including type I and IV collagen, TIMP-1, and MMPs (MMP-2 and MMP-9). One potential mechanism involves the inhibition of the TGF-β/SMAD pathway, as demonstrated by a decrease in *SMAD2/3* transcription factor levels. Additionally, modulation of the Wnt/β-catenin signaling pathway is implicated, supported by downregulation of the *LPR5* receptor and protein kinase cAMP-activated catalytic subunit alpha. In this study, the possible anti-fibrotic properties of 1-PHE are first demonstrated. To completely comprehend the effects of this substance in the context of the intricacies of liver fibrosis, experimental binding validation and in vivo investigations are necessary.

## Figures and Tables

**Table 1:** The list of top 10 up- and downregulated proteins of LX-2 cells in response to 1-PHE treatment.

Uniprot ID	Gene ID	Description	Log2 Fold Change	−log10 *p*-Value
Top 10 upregulated proteins				
O15355	*PPM1G*	Protein phosphatase	4.30	4.29
Q12904	*AIMP1*	Aminoacyl tRNA synthase complex-interacting multifunctional protein 1	2.95	3.16
Q16537	*PPP2R5E*	Serine/threonine-protein phosphatase 2A 56 kDa regulatory subunit epsilon isoform	2.86	1.42
Q16186	*ADRM1*	Proteasomal ubiquitin receptor ADRM1	2.84	2.32
P04066	*FUCA1*	Tissue alpha-L-fucosidase	2.80	1.92
O00139	*KIF2A*	Kinesin-like protein KIF2A	2.68	2.46
Q9UNF0	*PACSIN2*	Protein kinase C and casein kinase substrate in neurons protein 2	2.56	1.89
Q9BRK5	*SDF4*	45 kDa calcium-binding protein	2.45	1.40
Q0VDF9	*HSPA14*	Heat shock 70 kDa protein 14	2.43	2.18
Q09161	*NCBP1*	Nuclear cap-binding protein subunit 1	2.42	1.64
Top-10 downregulated proteins				
O75197	*LRP5*	Low-density-lipoprotein-receptor-related protein 5	−2.28	4.03
Q15582	*TGFBI*	Transforming growth factor-beta-induced protein ig-h3	−2.20	1.42
P04264	*KRT1*	Keratin, type II cytoskeletal 1	−1.97	2.04
Q9Y266	*NUDC*	Nuclear migration protein nudC	−1.91	3.85
Q92522	*H1FX*	Histone H1x	−1.87	5.21
P22087	*FBL*	rRNA 2′-O-methyltransferase fibrillarin	−1.87	3.35
Q9BS40	*LXN*	Latexin	−1.65	1.83
Q5SRE5	*NUP188*	Nucleoporin NUP188 homolog	−1.63	2.46
P07858	*CTSB*	Cathepsin B	−1.60	2.06

**Table 2:** The molecular docking analysis of selected proteins and compounds.

Protein Name	Ligand	AutoDock Vina Score	Interacting Amino Acid
Protein kinase cAMP-activated catalytic subunit alpha (PRKACA)	1-PHE	−6.033	Val57, Ala70, Glu127, Leu173
	3SB	−6.306	Leu49, Val57, Ala70, Lys72, Asp166, Asn171, Leu173, Thr183, Asp184
Low-density-lipoprotein-receptor-related protein 5 (LRP5)	1-PHE	−6.055	Ala454, Ile455, Ala553, Val555, Ala598, Val600

**Table 3:** The oligonucleotide primer pairs used in this study.

Gene ID	Description	Forward (5′ → 3′)	Reverse (5′ → 3′)
*TIMP1*	TIMP metallopeptidase inhibitor 1	CAAGATGTATAAAGGGTTCCAAGC	TCCATCCTGCAGTTTTCCAG
*MMP2*	Matrix metallopeptidase 2	AAGTATGGCTTCTGCCCTGA	ATTTGTTGCCCAGGAAAGTG
*COL1A1*	Collagen type I alpha 1 chain	CCGGCTCCTGCTCCTCTTAGCG	CGTTCTGTACGCAGGTGATTGGTGG
*COL4A1*	Collagen type IV alpha 1 chain	CCTGGCTTGAAAAACAGCTC	CCCTGCTGAGGTCTGTGAAC
*SMAD2*	SMAD family member 2	TGCTCTGAAATTTGGGGACTGA	GACGACCATCAAGAGACCTGG
*SMAD3*	SMAD family member 3	ATCGTGAAGCGCCTGCTG	CATCCAGGGACCTGGGGA
*GAPDH*	Glyceraldehyde-3-phosphate dehydrogenase	ATGACATCAAGAAGGTGGTG	CATACCAGGAAATGAGCTTG

## Data Availability

The original data presented in the study are openly available in ProteomeXchange Consortium via the PRIDE partner repository at http://www.ebi.ac.uk/pride/archive/PXD052809 (accessed on 4 June 2024). The following username and password can be used to access the dataset by logging in to the PRIDE website: Username: reviewer_pxd052809@ebi.ac.uk. Password: WQ6fuT3osQOJ.
